# Supplementary material for: Experimental realization of temporal refraction and reflection in elastic beams
Source: Nat Commun. 2025 Oct 28;16:9520. doi: 10.1038/s41467-025-64530-8 (PMC12568997; doi:10.1038/s41467-025-64530-8)
Supplement: Supplementary file 1 — Supplementary Information [file 41467_2025_64530_MOESM1_ESM.pdf]

Supplementary Information: Experimental Realization of Temporal Refraction and Reflection in Elastic Beams

Shaoyun Wang,[1,*] Nan Shao,[1,*] Hui Chen,[2] Jiaji Chen,[1] Honghua Qian,[1]
Qian Wu,[1] Huilin Duan,[3] Andrea Alù,[4,5,†] and Guoliang Huang[3,‡]

[1] *Department of Mechanical and Aerospace Engineering, University of Missouri, Columbia, MO 65211, USA*
[2] *Center for Mechanics Plus under Extreme Environments, School of Mechanical Engineering and Mechanics, Ningbo University, Ningbo 315211, China*
[3] *Department of Mechanics and Engineering Science, College of Engineering, Peking University, Beijing 100871, PR China*
[4] *Photonics Initiative, Advanced Science Research Center, City University of New York, New York, NY, USA*
[5] *Physics Program, Graduate Center, City University of New York, New York, NY, USA*

(Dated: August 27, 2025)

CONTENTS

1. Determine the effective bending stiffness from numerical tests 2

2. Experimental details 2
   A. Geometric and material parameters of the metabeam 2
   B. Experiment layout and measurement setup 4

3. Verification of temporal reflection in the experiment 5

4. Numerical simulation of temporal refraction and reflection 6
   A. Numerical simulation of temporal refraction and reflection in a metabeam 6
   B. Numerical simulation of temporal refraction and reflection in a long metabeam 7

5. Refraction and reflection at a time interface for the switch from OFF to ON in experiment 8

6. Refraction and reflection at a time interface for different frequencies in the experiment 9

7. Numerical investigation of the effect of finite switching time 10

8. Numerical study of temporal refraction and reflection of an asymmetric pulse 10

9. Derivation of temporal continuity conditions 11

10. Relationship between the angles of incidence and refraction 12

11. Justification of the Euler–Bernoulli Model: Timoshenko Effects, Mode Truncation, Damping, and Nonlinearity 12

12. Noether's theorem and conservation laws 15
    A. Complex scalar field theory for the Euler-Bernoulli beam 15
    B. Time translation symmetry and energy conservation 17
    C. Space translation symmetry and momentum conservation 18

13. Optimization method for broadband anti-reflection and wave amplification at multiple time interfaces 19

14. Resistor functions in smart waveform morphing and information 19

References 20

* These two authors contributed equally.
† Corresponding author: aalu@cuny.edu
‡ Corresponding author: guohuang@pku.edu.cn

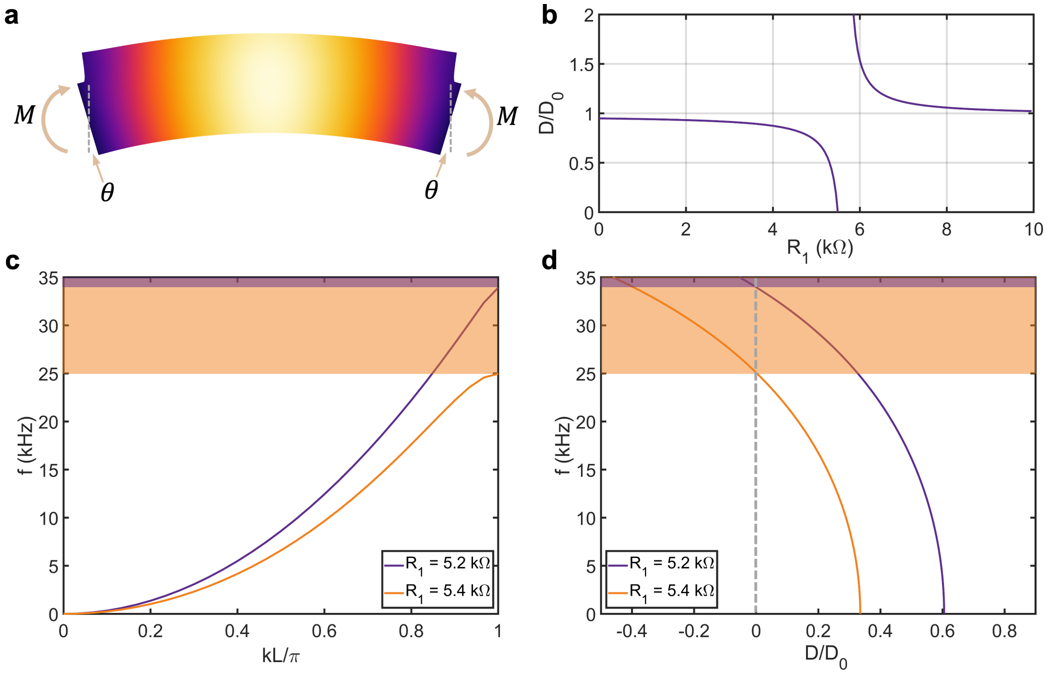

FIG. S1. **The effective bending stiffness and its application**. **a** Setup for determining the effective stiffness in the numerical test. **b** The relationship between normalized effective stiffness and R_1. D_0 represents the bending stiffness for the open circuit. **c** The dispersion curves for different values of R_1. The purple region indicates the bandgap for $R_1 = 5.2$ kΩ, while the orange region indicates the bandgap for $R_1 = 5.4$ kΩ. **d** The normalized stiffness as a function of frequency. The purple region shows the negative stiffness area for $R_1 = 5.2$ kΩ, and the orange region shows the negative stiffness area for $R_1 = 5.4$ kΩ.

1. DETERMINE THE EFFECTIVE BENDING STIFFNESS FROM NUMERICAL TESTS

A schematic diagram of the numerical test is shown in Fig. S1a. To determine the effective bending stiffness, the rotational angles at the left and right boundaries of the metabeam unit cell are set to $-\theta$ and θ, respectively, using rigid connectors in COMSOL. The vertical displacements at both boundaries are constrained to zero, while the horizontal displacements are left free. The reaction bending moment M at each rigid connector is obtained for calculating the effective bending stiffness. By solving the problem in the frequency domain, the effective bending stiffness D of the metabeam is defined following the approach described by Chen [**?**]:

$$D = \frac{M}{2\theta/L}, \tag{S1}$$

where L is the length of the unit cell. The normalized effective bending stiffness as a function of the resistor R_1 is shown in Fig. S1b. For R_1 values smaller than 5.5 kΩ, the normalized stiffness decreases to zero as R_1 increases, demonstrating the potential for tuning the bending stiffness using the negative capacitance circuit. For temporal refraction and reflection, $R_1 = 5$ kΩ, resulting in a normalized stiffness of $D/D_0 = 0.72$ at the operating frequency of 6 kHz, where $D_0 = 0.88\,\mathrm{N\cdot m^2}$. In general, the bending stiffness is frequency-dependent, and negative bending stiffness indicates the presence of bandgaps. As shown in Fig. S1c,d, the range of bandgaps corresponds to the range of negative normalized effective stiffness for different R_1 values. Additionally, the average density of the metabeam is $4143\,\mathrm{kg/m^3}$, and $\rho A = 0.116\,\mathrm{kg/m}$.

2. EXPERIMENTAL DETAILS

A. Geometric and material parameters of the metabeam

The metabeam consists of 30 unit cells, each equipped with a piezoelectric patch connected to a negative capacitance circuit via an analog switch, along with an additional unit cell used for excitation, as illustrated in Fig. S2a. The 30 piezoelectric patches are bonded to the aluminum beam using conductive epoxy, which is cured at room temperature for 24 hours. The spacing between adjacent piezoelectric patches is 0.67 mm. The geometric and material parameters of the metabeam, shown in Fig. S2b, are summarized in Table S1.

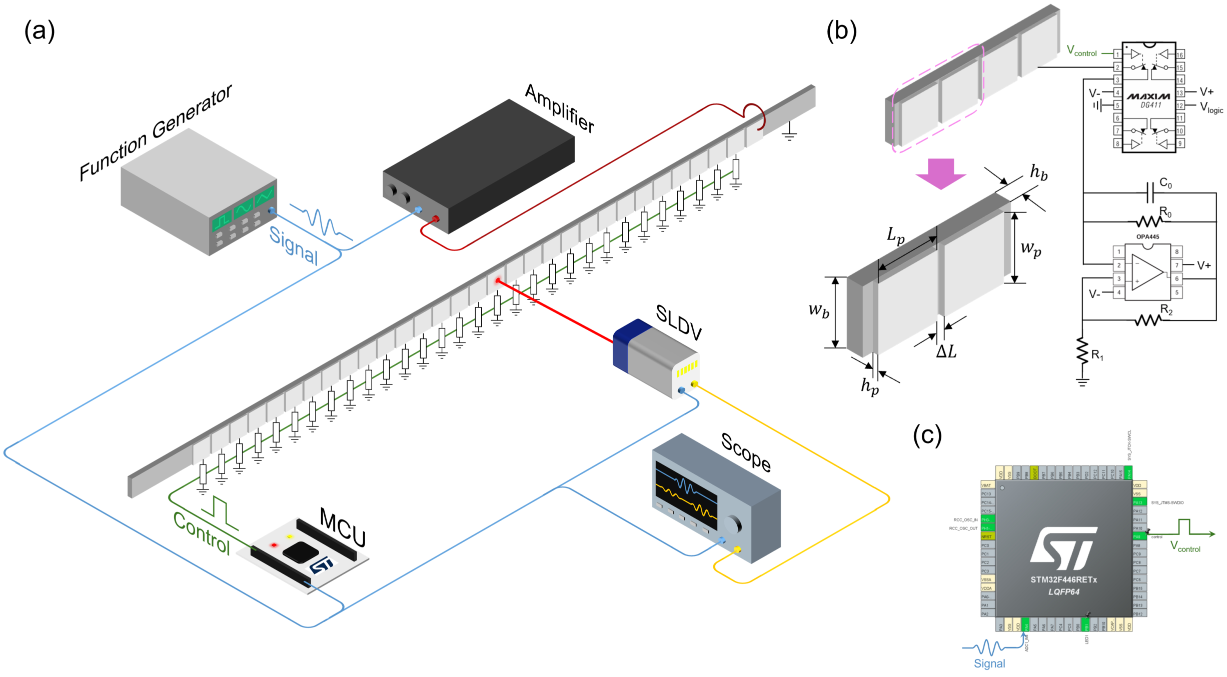

FIG. S2. **Practical experiment layout and measurement setup**. **a** Simplified schematic of the experimental setup. A signal generated by a function generator is amplified by a power amplifier to produce elastic waves through a piezoelectric patch. This signal also triggers an MCU to control all analog switches and activates the scanning laser Doppler vibrometer (SLDV) to measure the transverse velocity of the metabeam's surface. **b** Electrical control circuit layout and schematic for a single unit cell, along with its geometric parameters. **c** MCU pinout view.

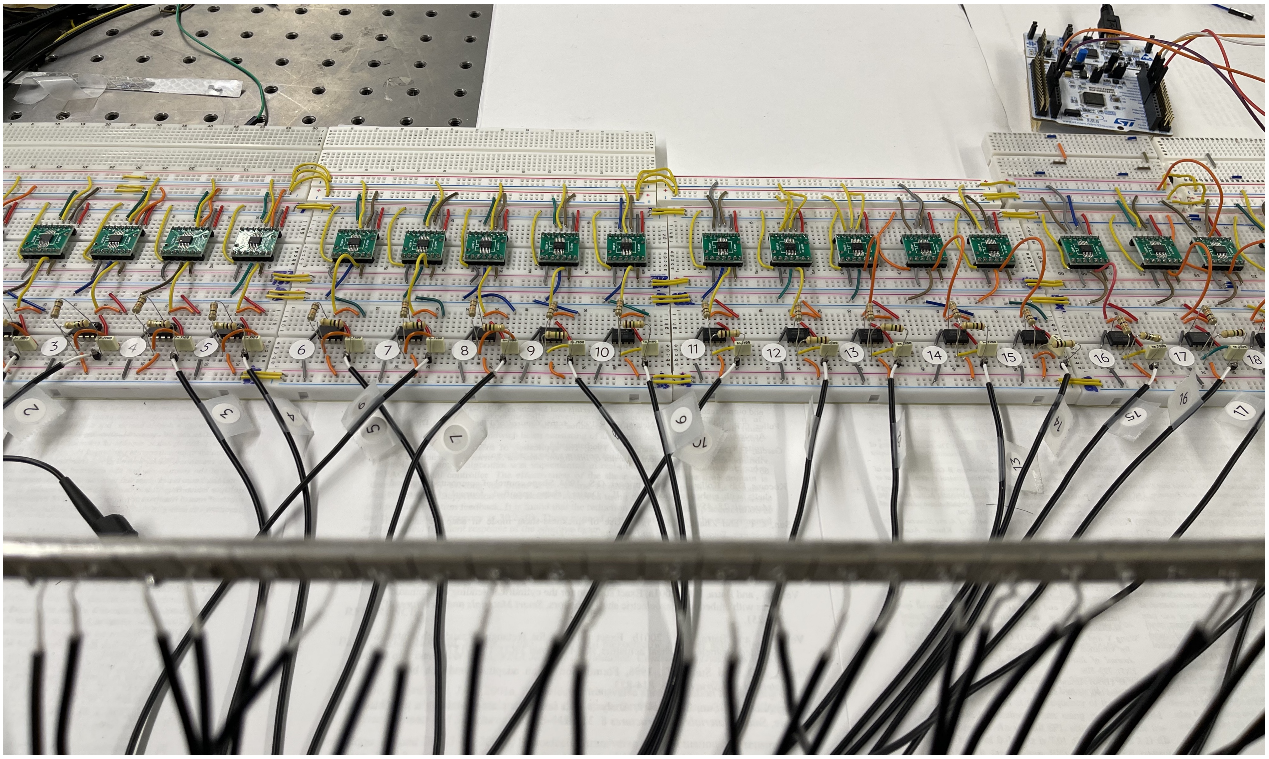

FIG. S3. **A photograph of metabeam with circuits**.

TABLE S1. **Geometric and material parameters of the metabeam.**

Parameter	Description	Value
h_b	Thickness of the host beam	2 mm
w_b	Width of the host beam	1 cm
L_p	Length of the piezoelectric patches	1 cm
h_p	Thickness of the piezoelectric patches	0.8 mm
w_p	Width of the piezoelectric patches	1 cm
ΔL	Interval between two piezoelectric patches	0.67 mm
E_b	Young's modulus of the aluminum beam	70 GPa
ρ_b	Density of the aluminum beam	2700 kg/m^3
s_{11}^E	Compliance matrix of the piezoelectric patches	1.64×10^{-11} 1/Pa
s_{33}^E	Compliance matrix of the piezoelectric patches	1.88×10^{-11} 1/Pa
s_{44}^E	Compliance matrix of the piezoelectric patches	4.75×10^{-11} 1/Pa
s_{66}^E	Compliance matrix of the piezoelectric patches	4.43×10^{-11} 1/Pa
s_{12}^E	Compliance matrix of the piezoelectric patches	-5.74×10^{-12} 1/Pa
s_{13}^E	Compliance matrix of the piezoelectric patches	-7.22×10^{-12} 1/Pa
d_{33}	Coupling matrix of the piezoelectric patches	3.74×10^{-11} C/N
d_{31}	Coupling matrix of the piezoelectric patches	-1.71×10^{-11} C/N
d_{15}	Coupling matrix of the piezoelectric patches	5.84×10^{-11} C/N
ϵ_{33}^S	Coupling matrix of the piezoelectric patches	$919.1\epsilon_0$
ϵ_{11}^S	Coupling matrix of the piezoelectric patches	$826.6\epsilon_0$
ϵ_0	Vacuum permittivity	8.842×10^{-12} F/m
ρ_p	Density of the piezoelectric patches	7750 kg/m^3

TABLE S2. **List of components used to fabricate metabeam, referred to the schematic shown in Fig. S2.**

Component	Description	Value
C_0	NC circuit capacitor	1 nF
R_0	NC circuit resistor	1 MΩ
R_1	NC circuit resistor	5 kΩ
R_2	NC circuit resistor	10 kΩ
Analog switch	DG411	
Operational amplifier	OPA445	
MCU	STM32F446	

B. Experiment layout and measurement setup

An electric signal generated by a function generator (Tektronix AFG3022C) is amplified by a power amplifier (Krohn-Hite) to excite elastic waves via the piezoelectric patch at rightmost. This signal simultaneously triggers a microcontroller unit (STM32F446RE) to control the analog switches and activates the scanning laser Doppler vibrometer (Polytec PSV-400) to measure the transverse velocity on the surface of the metabeam, as illustrated in Fig. S2a. A photograph of the

TABLE S3. **The angles of the incident wave, refracted wave, and reflected wave.**

Cases	α_0	α_1	α_1'
ON-to-OFF at 6 kHz	49.0°	52.0°	52.5°
ON-to-OFF at 6 kHz	48.0°	43.0°	43.5°
ON-to-OFF at 8 kHz	40.5°	43.5°	43.5°
OFF-to-ON at 8 kHz	45.0°	40.0°	40.0°
ON-to-OFF at 10 kHz	42.0°	45.5°	45.0°
OFF-to-ON at 10 kHz	42.0°	39.0°	39.5°

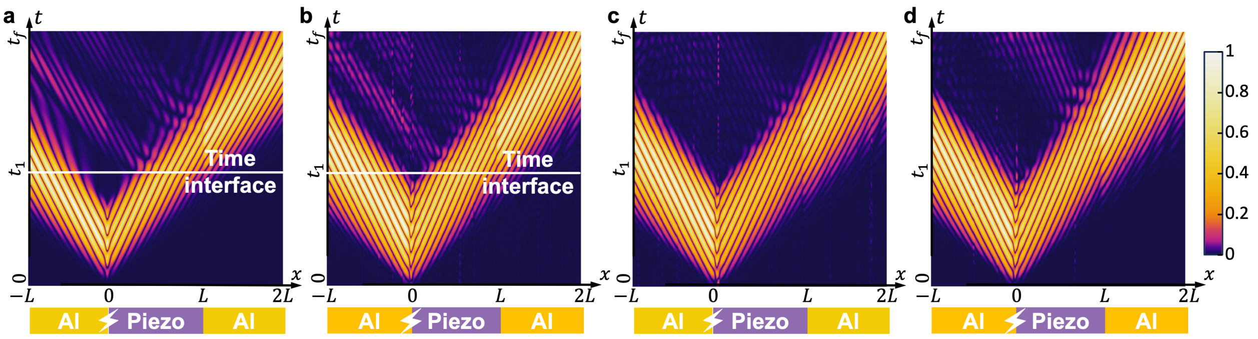

FIG. S4. **Spacetime diagram of temporal refraction and reflection without masks**. **a**, **b** The top panels show the simulation (**a**) and experimental results (**b**) of wave scattering from an incident wave packet consisting of 3 cycles in the time domain, with parameters $t_f = 1.1\,\mathrm{ms}$ and $t_1 = 0.44\,\mathrm{ms}$, at a time interface in a spacetime diagram. The bottom panels depict the system configuration, consisting of a length $L = 0.32\,\mathrm{m}$, composed of two aluminum beams (Al) and a piezo-metabeam (Piezo). The excitation source (indicated by the lightning symbol) is positioned at the left interface. **c**, **d** The spacetime diagrams illustrate the wave evolution with the switch permanently OFF (**c**) and permanently ON (**d**).

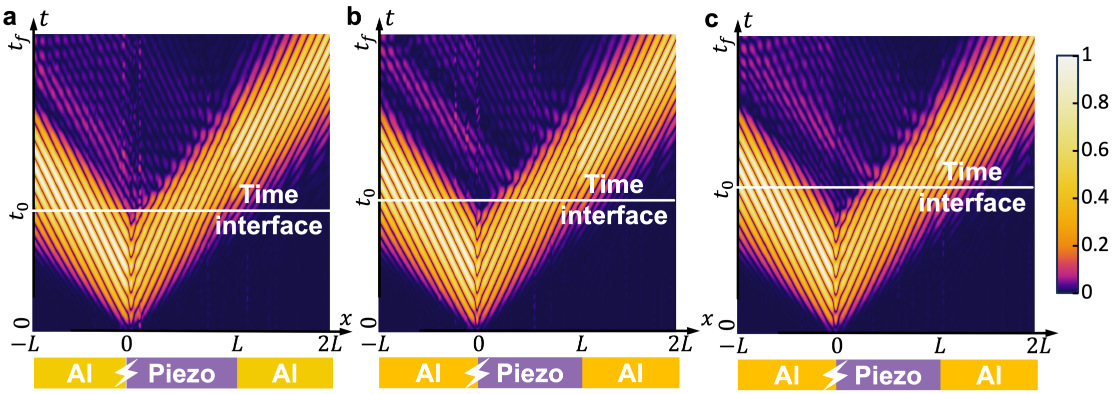

FIG. S5. **Temporal refraction and reflection at different switching times**. **a**, **b**, and **c** show the spacetime diagrams of wave evolution for switching times $t_1 = 0.435$ ms, $t_1 = 0.44$ ms, and $t_1 = 0.445$ ms, respectively.

experimental setup, showing the metabeam and its circuits, is presented in Fig. S3.

The laser Doppler vibrometer records data for 25.6 ms at a sampling rate of $f_s = 1.28$ MHz. The scanned domain, spanning 1.38 m, is discretized into a grid of 219 evenly spaced points, achieving a spatial resolution of 0.0063 m. Fig. S2b provides a schematic of the electrical control system used in each unit cell. The connection status between the piezoelectric patches and the negative capacitance circuits is controlled by analog switches, allowing adjustment of the effective stiffness of the metabeam. When V_{control} is at a high voltage level, the switch is OFF, and it is ON at a low voltage level. The switching time is less than 150 ns for transitions from OFF to ON and less than 100 ns for transitions from ON to OFF. These times are approximately 1000 times shorter than the bending wave period (about 150 µs), creating an ideal temporal interface.

All electrical circuits are constructed on solderless breadboards. The pinout of the MCU is shown in Fig. S2c. The MCU receives the reference signal through the analog-to-digital converter (ADC) at a sampling rate of 2 MHz. Once the voltage of the reference signal exceeds a specified threshold (−2 V), the MCU changes the state of the analog switch after a precisely programmed delay. The circuit parameters and components are detailed in Table S2.

3. VERIFICATION OF TEMPORAL REFLECTION IN THE EXPERIMENT

In Fig. 2a,c of the main text, masks were added to highlight the temporally reflected wave. Here, the masks are removed, and the original figures are presented in Fig. S4a,b. Since the behavior of spatially reflected waves, caused by spatial inhomogeneity, closely resembles that of temporally reflected waves, it is crucial to verify that the reflected waves in Fig. S4a,b are indeed temporally reflected waves rather than spatially reflected waves.

First, we perform a numerical simulation using the same setup as the experiment. The wave evolution in the spacetime diagram from the simulation is shown in Fig. S4a. Since the excitation is applied at the left interface between the aluminum beam and the piezoelectric metabeam, both a left-propagating wave and a right-propagating wave are generated. When the right-propagating wave reaches the right interface between the piezoelectric metabeam and the aluminum beam, a reflected wave is produced due to the spatial interface. In the simulation, an additional reflected wave is generated at the

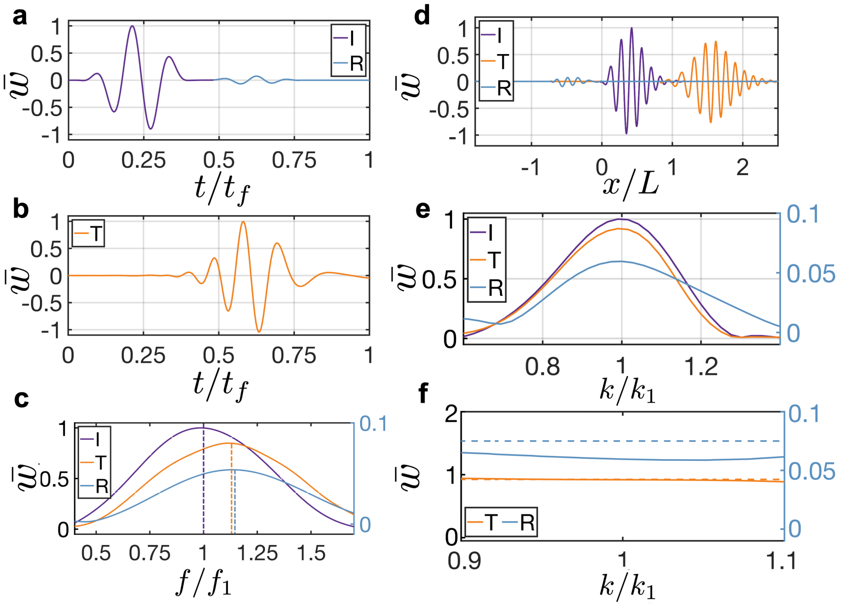

FIG. S6. **Spectral analysis of wave refraction and reflection at a time interface based on numerical simulation data.** **a** The incident (purple) and reflected (blue) signals measured at $x = 0.05L$. **b** The refracted signal (orange) observed at $x = L$. **c** Spectral analysis of the time-domain signals from (**a**, **b**). **d** The incident spatial profile (purple) measured at $t = 1/3t_f$, and the reflected and transmitted spatial profiles (blue and orange, respectively) measured at $t = 2/3t_f$. **e** Spectral analysis of the spatial-domain signals from (**d**). **f** The normalized spectral data for the incident, reflected, and refracted waves from (**e**), with each normalized by the amplitude distribution of the incident wave.

time interface, which is distinct from both the left-propagating wave caused by the excitation and the spatially reflected wave. This temporally reflected wave, also observed in the experiment (see Fig. S4b), closely resembles the one in the simulation, suggesting that it is indeed the time-reflected wave.

In the experiment, the piezoelectric metabeam consists of 30 unit cells, making it challenging to achieve perfect homogeneity across all cells. This inhomogeneity can potentially induce spatially reflected waves within the metabeam. To rule out this possibility, the spacetime diagrams of wave evolution with the switch permanently OFF and ON are presented in Fig. S4c,d for comparison. In these figures, no spatially reflected waves are observed in the middle of the metabeam, indicating that the metabeam is homogeneous. The homogeneity of the metabeam is ensured through the following steps. When the switch is OFF, homogeneity is maintained by carefully attaching the piezoelectric patches at constant intervals. When the switch is ON, resistors with $R_1 = 5$ kΩ are initially used, and the spacetime diagram is measured. While the metabeam is generally homogeneous, a few unit cells exhibit stiffness variations, leading to spatially reflected waves visible in the diagram. These abnormal cells are identified, and their resistors are replaced with potentiometers. By tuning the potentiometers until the reflected signals disappear from the PSV-400 screen, uniformity is achieved.

Furthermore, a key characteristic of a temporally reflected wave is that its position depends on the timing of the time interface. To verify this, we conduct three experimental tests with switching times of $t_1 = 0.435$ ms, 0.44 ms, and 0.445 ms, as shown in Fig. S5. In these figures, the position of the reflected wave shifts with changes in the time interface, confirming that the reflected wave depends on the time interface. This observation indicates that it is a temporally reflected wave rather than a spatially reflected one.

Based on these observations, we conclude that the reflected wave is a temporally reflected wave rather than a spatially reflected wave.

4. NUMERICAL SIMULATION OF TEMPORAL REFRACTION AND REFLECTION

A. Numerical simulation of temporal refraction and reflection in a metabeam

In Fig. 2(e–h), the temporal and spatial slices, along with their Fourier transforms, of the experimental spacetime diagram shown in Fig. 2(a) are presented. However, the corresponding results for the simulated spacetime diagram in Fig. 2(c) are not provided. Here, the corresponding simulation results are shown in Fig. S6 for comparison. The simulation results in Fig. S6 closely align with the experimental results in Fig. 3 of the main text. The frequency of the refracted and reflected waves is approximately 1.13 f_0, which is close to the theoretical value of 1.17 f_0. Furthermore, the normalized amplitude of the reflected wave with respect to the wave number is flatter and closely matches the theoretical prediction, indicating that the amplitude relation predicted in Eq. (9) of the main text holds over a wide frequency range.

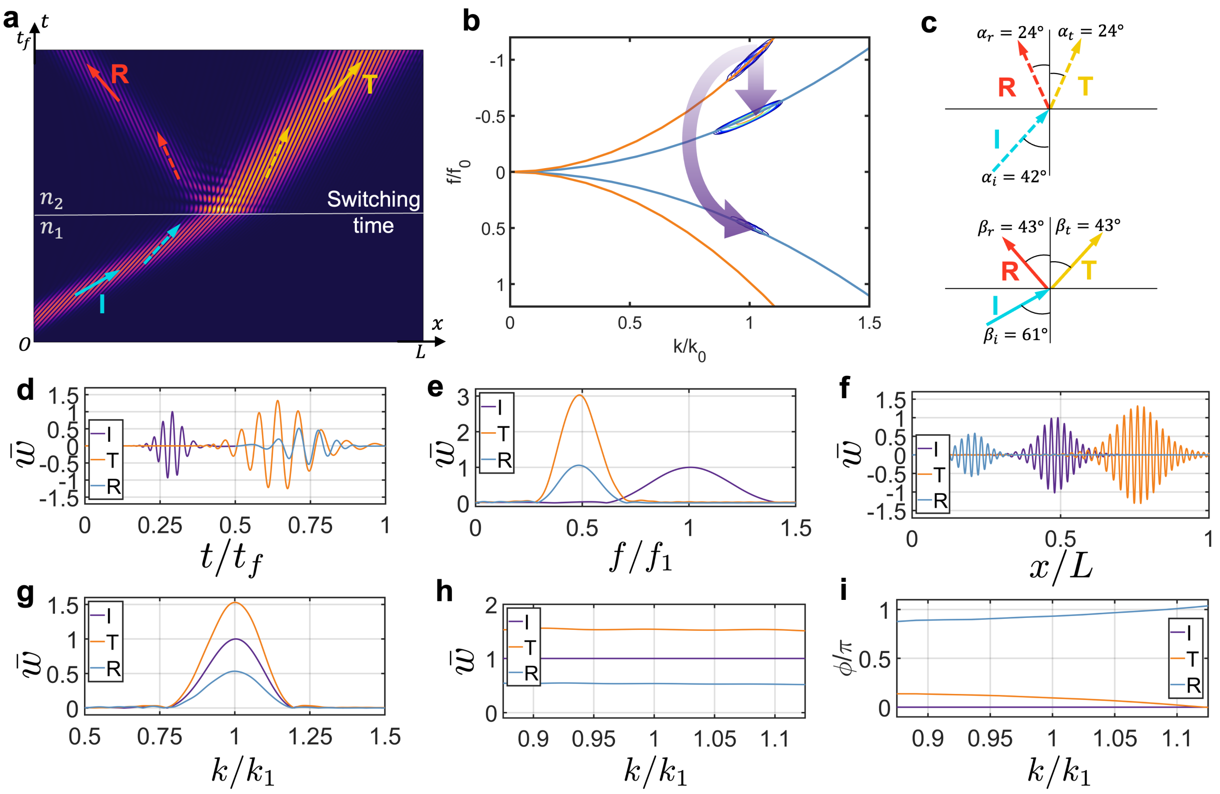

FIG. S7. **Numerical investigation of temporal refraction and reflection in a long metabeam.** **a** The refraction and reflection process of an incident wave packet, consisting of 5 cycles in the time domain, visualized in a spacetime diagram. Here, $L = 2.56\,\mathrm{m}$ and $t_f = 6\,\mathrm{ms}$. **b** The dispersion curves of the medium before (orange) and after (blue) the switching event, overlaid with a background contour diagram obtained from a 2D Fourier transform of the spacetime data in **a**. **c** The angular relationship between the incident, reflected, and refracted waves for both a plane wave and a wave packet, with arrows translated from the spacetime diagram in **a**. **d** The incident (purple) and reflected (blue) signals measured at $x = 0.28L$, and the refracted signal (orange) observed at $x = 0.62L$. **e** The spectral analysis of the time-domain signals in **d**. **f** The incident spatial profile (purple) measured at $t = 0.25t_f$, along with the reflected and transmitted spatial profiles (blue and orange, respectively) measured at $t = 0.75t_f$. **g** The spectral analysis of the spatial-domain signals in **f**. **h** The normalized spectral data for the incident, reflected, and refracted waves in **g**, each normalized by the amplitude distribution of the incident wave. **i** The relationship between phase and wavenumber.

B. Numerical simulation of temporal refraction and reflection in a long metabeam

In the experiment, the metabeam's normalized stiffness can be reduced to a minimum of 0.72, resulting in a relatively small reflected wave. To better observe temporal refraction and reflection, we perform a simulation using a long metabeam with 240 unit cells, excited by a 5-cycle tone burst. In the simulation, the switch transitions from OFF to ON, with the bending stiffness in the ON state being one-quarter of that in the OFF state. Under these conditions, the refractive index ratio is $n_1/n_0 = 2$, and the impedance ratio is $Z_1/Z_0 = 1/2$.

In Fig. S7a, the incident wave splits into a refracted wave and a reflected wave upon encountering the time interface. A 2D Fourier transform is applied to the data in Fig. S7a, with the results shown in Fig. S7b. In Fig. S7b, the frequency of the incident wave shifts from f_0 to $f_0/2$ and $-f_0/2$, while the wavenumber remains constant. In Fig. S7c, the directions of the incident, refracted, and reflected plane waves (solid arrows) and wave packets (dashed arrows) are derived from their respective components in Fig. S7a. The incident angle of the plane waves is $\alpha_i = 42°$, with refracted and reflected angles of $\alpha_t = 24°$ and $\alpha_r = 24°$, respectively. The ratio $\tan\alpha_t/\tan\alpha_i = 2.02$ closely matches the refractive index ratio $n_1/n_0 = 2$, verifying Snell's law in Eq. (S12) of the main text. For the wave packet, the incident angle is $\beta_i = 61°$, while the refracted and reflected angles are $\beta_t = 43°$ and $\beta_r = 43°$, respectively. The ratio $\tan\beta_t/\tan\beta_i = 1.93$ is close to the refractive index ratio, validating the geometric relationship of the wave packet described in Eq. (S13) of the main text.

Next, we validate the Fresnel equations presented in Eq. (9) of the main text. In Fig. S7d, the incident wave splits into a refracted wave and a reflected wave in the time domain. In the frequency domain, as shown in Fig. S7e, the frequencies of the refracted and reflected waves are approximately half that of the incident wave, quantitatively confirming the frequency shift after passing through the temporal interface, as described in Eq. (7) of the main text. In Fig. S7f, the incident wave splits into a refracted wave and a reflected wave in the spatial domain. The spectral data from Fig. S7f is presented in Fig. S7g. The wavenumbers of the waves remain constant, indicating momentum conservation. The peak amplitude

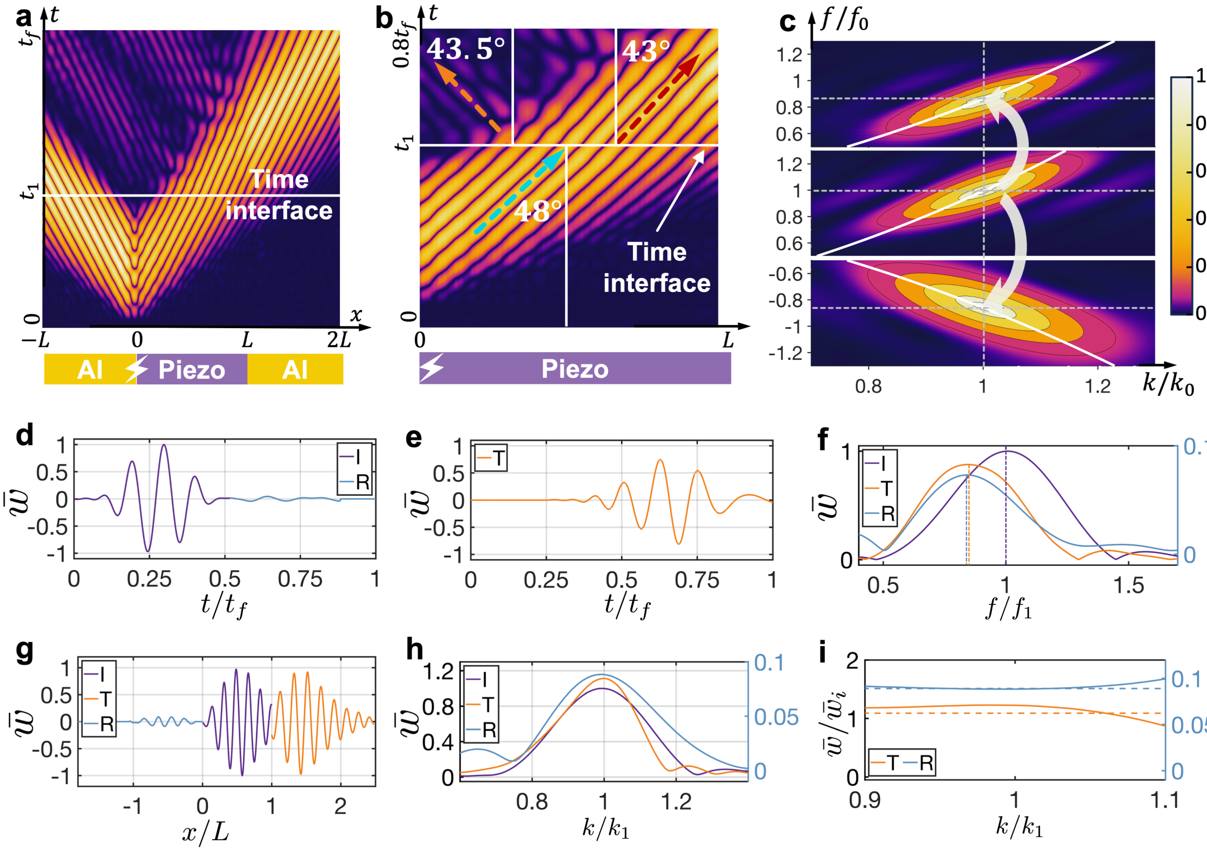

FIG. S8. **Temporal refraction and reflection in the metabeam with switch from OFF to ON.** **a** (**b**) The top panels show the simulation (**a**) and experiment (**b**) of wave scattering from an incident wave packet consisting of 3 cycles in the time domain, with $t_f = 1.1\,\mathrm{ms}$ and $t_1 = 0.44\,\mathrm{ms}$, at a time interface in a spacetime diagram. The bottom panels depict the system, with $L = 0.32\,\mathrm{m}$, composed of two aluminum beams (Al) and a piezo-metabeam (Piezo), with the excitation (lightning symbol) located at the left interface. **c** The 2D Fourier transform of the experimental data shown in **b**. **d** The incident (purple) and reflected (blue) signals measured at $x = 0.05L$. **e** The refracted signal (orange) observed at $x = L$. **f** The spectral analysis of the time-domain signals from **d** and **e**. **g** The incident spatial profile (purple) measured at $t = 1/3t_f$, along with the reflected and transmitted spatial profiles (blue and orange, respectively) at $t = 2/3t_f$. **h** The spectral analysis of the spatial-domain signals from **g**. **i** The normalized spectral data for the reflected and refracted waves in **h**, each normalized by the amplitude distribution of the incident wave.

shifts from 1 in the incident wave to 1.5 in the refracted wave and 0.5 in the reflected wave, aligning with the results calculated using the Fresnel equations in Eq. (9) of the main text.

Furthermore, Fig. S7h presents the normalized amplitude distributions of the incident, transmitted, and reflected waves, with each normalized by the amplitude of the incident wave. This confirms that the amplitude ratio is independent of the wavenumber (frequency), as predicted by Eq. (7) of the main text. Additionally, the phase factors are deduced from the wave packet dynamics shown in Fig. S7i. The phase of the transmitted wave remains unchanged relative to the incident wave, while the phase of the reflected wave shifts by π relative to the incident wave across different wavenumbers, consistent with Eq. (9) of the main text.

5. REFRACTION AND REFLECTION AT A TIME INTERFACE FOR THE SWITCH FROM OFF TO ON IN EXPERIMENT

In this section, we examine temporal refraction and reflection during a switch transition from OFF to ON for an incident wave with a frequency of 6 kHz. The setup and parameters are identical to those in Fig. 2 of the main text. In this scenario, the refractive index ratio is $n_1/n_0 = 1.17$, and the impedance ratio is $Z_1/Z_0 = 0.85$. In Fig. S8a, the incident wave splits into a refracted wave and a reflected wave after passing through the time interface. In Fig. S8b, the incident angle of the plane waves is $\alpha_i = 48°$, with refracted and reflected angles of $\alpha_t = 43°$ and $\alpha_r = 43.5°$, respectively. The ratio $\tan\alpha_t/\tan\alpha_i = 0.84$ closely matches the refractive index ratio $n_1/n_2 = 0.85$, validating Snell's law as described in Eq. (S12) of the main text.

A 2D Fourier transform is applied to the data in Fig. S8a, with the results shown in Fig. S8c. In Fig. S8b, the frequency of the incident wave shifts from f_0 to $0.83f_0$ and $-0.83f_0$, while the wavenumber remains constant. To further confirm the frequency conversion and wavenumber invariance, time-domain signals measured at $x/L = 0.05$ and $x/L = 1$

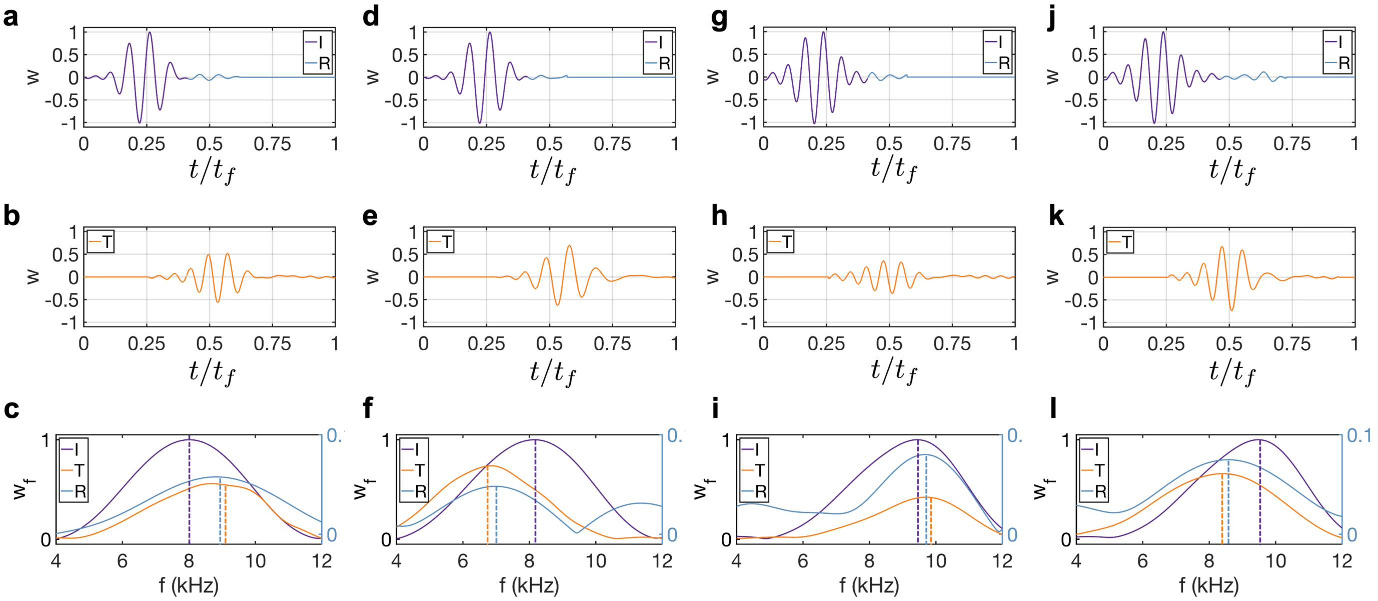

FIG. S9. **Spectral analysis of wave refraction and reflection at a time interface across different frequencies.** **a** (**d**) The incident temporal profile (purple) with an excitation frequency of 8 kHz, measured at $t = 1/3t_f$, and the reflected temporal profile (orange) at $t = 2/3t_f$. The switch transitions from ON to OFF (**a**) and from OFF to ON (**d**). **b** (**e**) The reflected and refracted temporal profiles (blue) at $t = 2/3t_f$. **c** (**f**) The spectral analysis of the incident, refracted, and reflected waves. **g** (**j**) The incident temporal profile (purple) with an excitation frequency of 10 kHz, measured at $t = 1/3t_f$, and the reflected temporal profile (orange) at $t = 2/3t_f$. The switch transitions from ON to OFF (**g**) and from OFF to ON (**j**). **h** (**k**) The reflected and refracted temporal profiles (blue) at $t = 2/3t_f$. **i** (**l**) The spectral analysis of the incident, refracted, and reflected waves.

are shown in Fig. S8d,e, where three distinct wave packets corresponding to the incident, refracted, and reflected waves are clearly visible. The normalized frequencies $f_t/f_0 = 0.83$ for the refracted wave and $f_r/f_0 = 0.83$ for the reflected wave quantitatively confirm the frequency shift relative to the incident wave, as shown in Fig. S8f, indicating a breakdown of energy conservation. Additionally, the spatial-domain signals measured at $t/t_f = 1/3$ and $t/t_f = 2/3$ are shown in Fig. S8g. The central wavenumbers k_t for the refracted wave and k_r for the reflected wave are consistent with the central wavenumber k_1 of the incident wave, as depicted in Fig. S8h, demonstrating the conservation of momentum. The normalized spectral data for the refracted and reflected waves is presented in Fig. S8i, where the normalized amplitudes are independent of the wavenumber and closely align with the theoretical predictions, thereby verifying the Fresnel equation in Eq. (9) of the main text.

6. REFRACTION AND REFLECTION AT A TIME INTERFACE FOR DIFFERENT FREQUENCIES IN THE EXPERIMENT

In this section, we examine wave refraction and reflection at different excitation frequencies, specifically 8 kHz (Fig. S9a–f) and 10 kHz (Fig. S9g–l), for switching transitions from ON to OFF and from OFF to ON, respectively. The excitation frequency refers to the frequency of the signal generated by the function generator, which may differ slightly from the incident frequency, defined as the peak position in the wave spectrum. The switching time in these cases is the same as that used for the case with an excitation frequency of 6 kHz.

As shown in Fig. S9c (f), the frequencies of the refracted and reflected waves shift from 8 kHz (incident wave) to 9 kHz and 7 kHz, respectively. In Fig. S9i (l), the frequencies of the refracted and reflected waves shift from 9.5 kHz (incident wave) to 9.8 kHz and 8.5 kHz, respectively.

For the 10 kHz case, the frequency shift does not satisfy Snell's law precisely. This discrepancy arises because the relatively high frequency leads to a smaller wavelength, increasing the ratio of the unit cell length to the wavelength. Consequently, the long-wave approximation is less valid, and the metabeam can no longer be considered a homogeneous beam. Furthermore, at higher frequencies, the homogeneous Euler-Bernoulli beam model becomes invalid, making the reflection and refraction less distinguishable and leading to inaccuracies in the frequency shift.

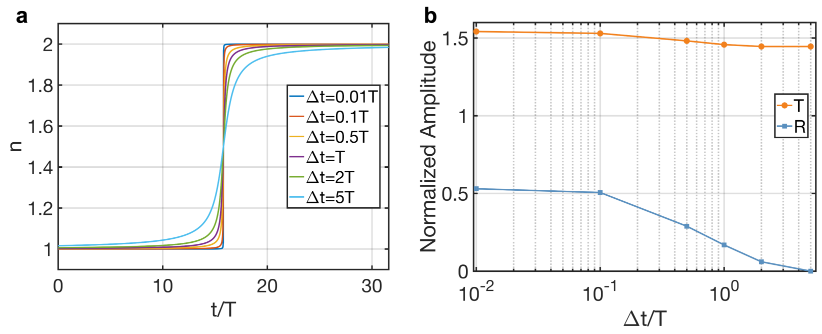

FIG. S10. **The effect of finite switching time.** **a** Different waveforms of $n(t)$ with parameters $\delta = 0.5$ and $\alpha = 1$. **b** Temporal refraction and reflection ratios as a function of $\Delta t/T$, where T is the period of the incident wave.

7. NUMERICAL INVESTIGATION OF THE EFFECT OF FINITE SWITCHING TIME

In the experiment, the switch does not transition instantaneously between states. In this section, we analyze the effect of finite switching time on the amplitudes of the refracted and reflected waves. To study this, various smooth time boundaries are modeled using the analytical function $n(t) = \frac{\Delta n}{\pi} \arctan\left(\frac{t-t_c}{\Delta t}\right) + n_0 + \frac{\Delta n}{2}$, where n_0 and $\Delta n = n_0$ represent the initial and changed refractive indices, and $\Delta t/T$ determines the sharpness of the waveform. Here, T is the period of the incident wave.

In Fig. S10a, the smooth step function is displayed for different values of $\Delta t/T$. The corresponding normalized amplitudes of the refracted and reflected waves, relative to the incident wave, are shown in Fig. S10b. The numerical setup in this section is the same as that described in Part B of Supplementary Section IV. When $\Delta t/T$ is very small (less than 0.1), the amplitudes of both the refracted and reflected waves remain constant and closely align with the theoretical predictions from Fresnel's formula. As long as $\Delta t/T$ stays within this range, the time interface can be regarded as ideal. In our experiment, $\Delta t/T = 0.001$, which is much smaller than 0.1, confirming that the interface behaves as an ideal time interface.

If $\Delta t/T$ exceeds 0.1, the amplitudes of both the refracted and reflected waves decrease as $\Delta t/T$ increases. However, the amplitude of the refracted wave approaches a finite value of $\sqrt{2}$, while the amplitude of the reflected wave diminishes to zero. This indicates that no reflected wave is generated when the refractive index changes very gradually. This behavior can be explained by the adiabatic theorem, which states that a system will remain in the same eigenstate if its parameters vary slowly enough. In the adiabatic limit, the wave's evolution can be described by [**? ?**]:

$$w(x,t) = \frac{A_i}{\sqrt{\omega(t)}} e^{ikx - \int_{t_0}^{t} \omega(t')dt'}. \tag{S2}$$

In this scenario, the wave remains a right-propagating wave, with its amplitude and frequency gradually changing over time and no reflected wave being generated. Since the ratio of the final frequency to the initial frequency is $\sqrt{2}$, the normalized amplitude of the refracted wave becomes $\sqrt{2}$, consistent with the results shown in Fig. S10b. Between the sudden-change limit and the adiabatic limit, the normalized amplitudes vary continuously as $\Delta t/T$ increases, falling within the range defined by these two extremes.

8. NUMERICAL STUDY OF TEMPORAL REFRACTION AND REFLECTION OF AN ASYMMETRIC PULSE

In this section, we demonstrate that the observed reflection is a temporal reflection rather than a spatial one. To highlight this distinction, we consider the scattering of an asymmetric pulse at a temporal interface. The simulation setup is identical to that described in Supplementary Section 4B. As shown in Fig. S11a, the spacetime diagram captures the behavior of the pulse upon encountering the interface.

Key differences between spatial and temporal reflections are evident in the order of pulse peaks. For spatial reflection, the higher peak of the incident pulse appears first, followed by the lower peak. In contrast, temporal reflection reverses this order: the lower peak arrives first, and the higher peak follows. This reversal in the time domain is shown in the normalized signals in Fig. S11b. Here, the incident ($\bar{w}_i$) and reflected ($\bar{w}_r$) signals, measured at $x/L = 0.3$, clearly demonstrate this inversion. In the spatial domain, the incident waves are shown in Fig. S11c. Fig. S11d displays the refracted ($\bar{w}_t$) and reflected ($\bar{w}_r$) signals, which are normalized and measured at $t/t_f = 0.3$. The reflected wave packet with the smaller peak appears first, followed by the reflected wave packet with the larger peak, which is opposite to the order observed in spatial reflection.

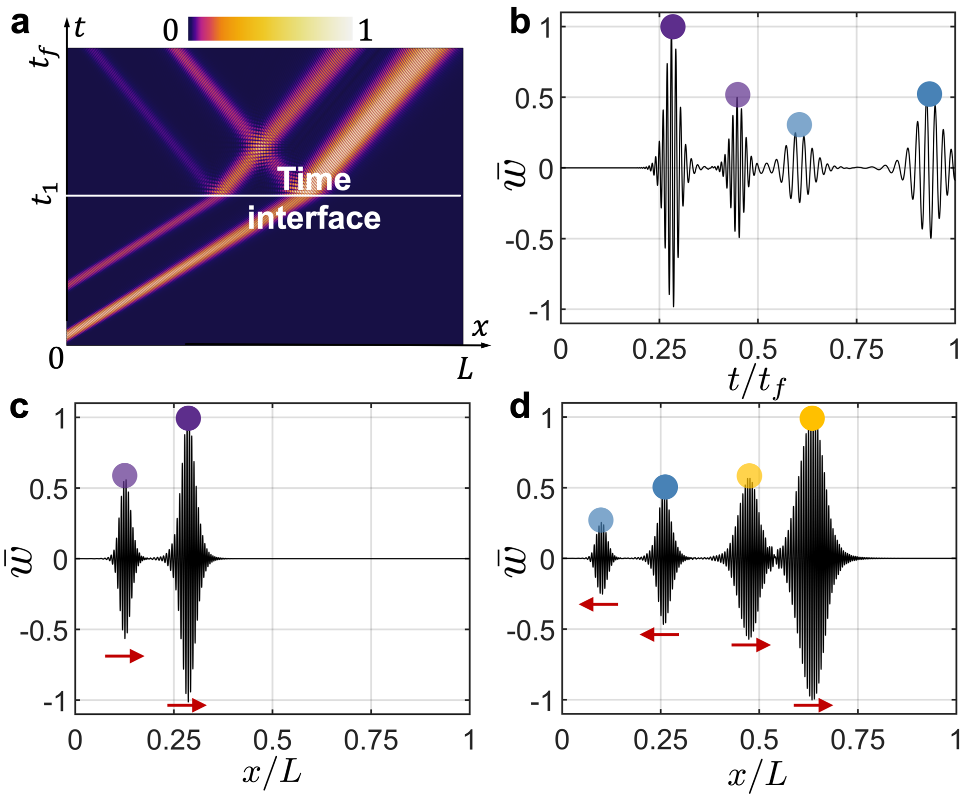

FIG. S11. **Refraction and reflection of an asymmetric pulse at a time interface.** **a** The spacetime diagram of a simulated asymmetric pulse scattering at a temporal interface. **b** The normalized incident ($\bar{w}_i$) and reflected ($\bar{w}_r$) signals measured at $x/L = 0.3$. The purple wave packet represents the incident wave with a higher amplitude, while the light purple wave packet corresponds to the incident wave with a lower amplitude. Similarly, the blue wave packet represents the reflected wave generated by the higher-amplitude incident wave, and the light blue wave packet corresponds to the reflected wave generated by the lower-amplitude incident wave. **c** The normalized incident ($\bar{w}_i$) signal measured at $t/t_f = 0.3$. The purple wave packet represents the incident wave with a higher amplitude, while the light purple wave packet corresponds to the incident wave with a lower amplitude. **d** The normalized refracted ($\bar{w}_t$) and reflected ($\bar{w}_r$) signals measured at $t/t_f = 0.3$. The orange wave packet represents the refracted wave with a higher amplitude, while the light orange wave packet corresponds to the incident wave with a lower amplitude. Similarly, the blue wave packet represents the reflected wave generated by the higher-amplitude incident wave, and the light blue wave packet corresponds to the reflected wave generated by the lower-amplitude incident wave.

9. DERIVATION OF TEMPORAL CONTINUITY CONDITIONS

The equation of motion for the Euler-Bernoulli beam is given by:

$$\partial_t p = \partial_{xx} M + q(t), \tag{S3}$$

where $p = \rho A \partial_t w$ represents the momentum, M is the bending moment, and q is a time dependent external force. Meanwhile, the bending curvature κ is defined as

$$\kappa = \partial_{xx} w. \tag{S4}$$

The bending momentum and bending curvature satisfy the constitutive relation

$$M = -D(t)\kappa. \tag{S5}$$

where $D(t) = E(t)I$ is the time-dependent bending stiffness, $E(t)$ is the time-dependent Young's modulus, and I it the second moment of area. Substituting Eq. (S5) and Eq. (S4) into the equation of motion (Eq. (S3)) yields the governing equations for the Euler-Bernoulli beam, as presented in Eq. (1) of the main text

$$\frac{\partial}{\partial t}\left(\rho A \frac{\partial w(x,t)}{\partial t}\right) + \frac{\partial^2}{\partial x^2}\left(D(t)\frac{\partial^2 w(x,t)}{\partial x^2}\right) = q(t). \tag{S6}$$

Integrating Eq. (S6) from initial time t' to an arbitrary time t gives

$$\rho A \frac{\partial w(x,t)}{\partial t}\Big|_{t'}^{t} + \int_{t'}^{t} dt D(t)\frac{\partial^4 w(x,t)}{\partial x^4} = \int_{t'}^{t} q(t)dt. \tag{S7}$$

Taking $t' = t_1^- = t_1 - \epsilon$ to $t = t_1^+ = t_1 + \epsilon$ with a $\epsilon \to 0^+$, we expect that the second term in Eq. (S7) is zero, due to the finite values of fields. The term on the right-hand side represents the impulse, which is not considered in our study and is therefore set to zero. Then, we obtain the first temporal boundary condition that describes the continuity of momentum:

$$\rho A \frac{\partial w}{\partial t}\Big|_{t=t_1^+} = \rho A \frac{\partial w}{\partial t}\Big|_{t=t_1^-}. \tag{S8}$$

Since the density is time-independent, Eq. (S8) implies the continuity of velocity

$$\frac{\partial w}{\partial t}\Big|_{t=t_1^+} = \frac{\partial w}{\partial t}\Big|_{t=t_1^-}. \tag{S9}$$

Similarly, integrating Eq. (S7) from t_1^- to t_1^+ as $\epsilon \to 0^+$ with respect to t without the impulse gives the second temporal boundary condition that describes the continuity of displacement:

$$w|_{t=t_1^+} = w|_{t=t_1^-}. \tag{S10}$$

10. RELATIONSHIP BETWEEN THE ANGLES OF INCIDENCE AND REFRACTION

In the main text, the Snell's law is written as

$$\omega_1 n_1 = \omega_0 n_0. \tag{S11}$$

The more familiar form describing the geometric relationship between the angles of the incident and refracted waves in a space-time diagram is presented as follows.

In the (x, ct) space, where c is a reference speed to maintain dimensional consistency, the angle α between the propagation direction of a plane wave (with phase velocity ω/k) and the time axis satisfies the relation $\tan\alpha = \omega/(kc) = k/(nc)$. This results in the geometric relationship between the angle of incidence and the angle of refraction:

$$\frac{\tan\alpha_0}{\tan\alpha_1} = \frac{n_1}{n_0}. \tag{S12}$$

Eq. (S12) can be interpreted as the temporal Snell's law, describing the geometric relationship between the angles of the incident and refracted waves in a space-time diagram. This is analogous to the traditional Snell's law, which applies to wave propagation in two-dimensional space.

The dispersion relation of flexural waves is a quadratic function, making them dispersive, meaning the propagation direction of a wave packet differs from that of a plane wave. We now explore the geometric relationship between the incident and refracted angles of the wave packet. The angle between the propagation direction of the wave packet and the time axis is defined as $\tan\beta = v_g/c$, where the group velocity $v_g = d\omega/dk = 2k\sqrt{D/\rho A} = 2k/n$. Therefore, the geometric relationship between the incident and refracted angles of the wave packet is

$$\frac{\tan\beta_0}{\tan\beta_1} = \frac{n_1}{n_0}. \tag{S13}$$

Interestingly, this relationship is the same as the temporal Snell's law for plane waves.

Fig. S12 shows the relationship between the angles of incidence and refraction in a space-time diagram. Here, the angle of the incident plane wave α_i, the angle of the refracted plane wave α_t, and the angle of the reflected plane wave α_r are $49°, 52°$, and $52.5°$, respectively. The ratios $\tan\alpha_i/\tan\alpha_t = 0.9$ and $\tan\alpha_i/\tan\alpha_r = 0.88$ are close to the refractive index ratio $n_1/n_0 = 0.85$, confirming the temporal Snell's law in Eq. (S12). The geometric relation of the wave packet in Eq. (S13) is verified in a longer metabeam using 5-cycle tone-burst excitation, as detailed in Supplementary Section 4B.

11. JUSTIFICATION OF THE EULER–BERNOULLI MODEL: TIMOSHENKO EFFECTS, MODE TRUNCATION, DAMPING, AND NONLINEARITY

For a Timoshenko beam, the coupled equations for the transverse displacement $w(x, t)$ and the cross-sectional rotation $\phi(x, t)$ are given by:

$$\begin{aligned} \rho A \frac{\partial^2 w}{\partial t^2} &= \frac{\partial}{\partial x}\left[\kappa AG\left(\frac{\partial w}{\partial x} - \phi\right)\right], \\ \rho I \frac{\partial^2 \phi}{\partial t^2} &= \frac{\partial}{\partial x}\left(EI\frac{\partial \phi}{\partial x}\right) + \kappa AG\left(\frac{\partial w}{\partial x} - \phi\right), \end{aligned} \tag{S14}$$

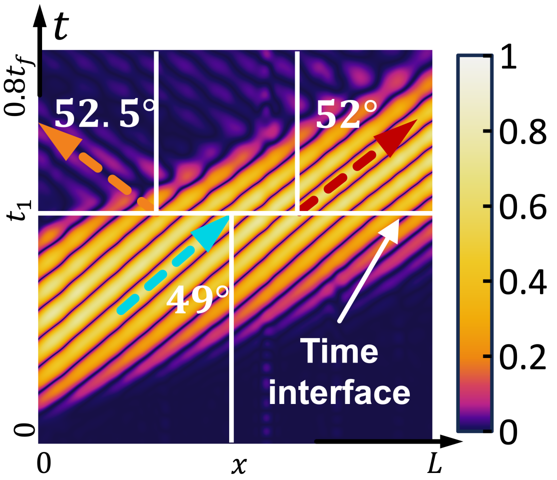

FIG. S12. **Relationship between the angles of incidence, refraction, and reflection**.

where ρ is the material density, A is the cross-sectional area, I is the area moment of inertia, E is Young's modulus, G is the shear modulus, and $\kappa = 5/6$ is the shear correction factor.

Assuming harmonic wave solutions of the form:

$$w(x,t) = We^{i(kx-\omega t)}, \quad \phi(x,t) = \Phi e^{i(kx-\omega t)}, \tag{S15}$$

the equations reduce to a compact matrix form:

$$\begin{pmatrix} \kappa AGk^2 - \omega^2\rho A & i\kappa AGk \\ -i\kappa AGk & EIk^2 + \kappa AG - \omega^2\rho I \end{pmatrix} \begin{pmatrix} W \\ \Phi \end{pmatrix} = \mathbf{0}. \tag{S16}$$

Setting the determinant of the coefficient matrix to zero yields the dispersion relation:

$$\frac{\rho^2 AI}{\kappa AG}\omega^4 - \left[\rho A + \left(\rho I + \frac{EI\rho A}{\kappa AG}\right)k^2\right]\omega^2 + EIk^4 = 0. \tag{S17}$$

Solving for ω as a function of k, we obtain the positive real branch:

$$\omega = \sqrt{\frac{\rho A + \left(\rho I + \frac{EI\rho A}{\kappa AG}\right)k^2 + \sqrt{\left[\rho A + \left(\rho I + \frac{EI\rho A}{\kappa AG}\right)k^2\right]^2 - 4\frac{\rho^2 AI}{\kappa AG}EIk^4}}{2\frac{\rho^2 AI}{\kappa AG}}}. \tag{S18}$$

The corresponding normalized eigenvector is given by:

$$\psi(\omega,k) = \frac{1}{\sqrt{(\kappa AGk^2 - \omega^2)^2 - (\kappa AGk)^2}} \begin{pmatrix} -i\kappa AGk \\ \kappa AGk^2 - \omega^2 \end{pmatrix}, \tag{S19}$$

and the complete time-harmonic solution becomes:

$$\Psi(x,t) = \psi(\omega,k)\, e^{i(kx-\omega t)}. \tag{S20}$$

Suppose that at a specific time $t = t_0$, the beam's effective stiffness changes abruptly due to the switching of an external electronic circuit. In our case, this change primarily affects the bending stiffness. We define the effective bending stiffness before and after the temporal interface as:

$$E_0 I \quad \text{and} \quad E_1 I, \tag{S21}$$

respectively. The piezoelectric patches do not affect the shear term or the mass parameters. Therefore, we assume that the shear term κAG and the mass parameters ρA and ρI remain unchanged.

Under this condition, Snell's law for the temporal interface can be expressed as:

$$\omega_0 n_0 = \omega_1 n_1, \tag{S22}$$

where n_0 and n_1 are the effective refractive indices corresponding to stiffnesses E_0I and E_1I, respectively, and are computed via Eq. (S18).

We assume the wavefield before the temporal interface takes the form:

$$\Psi_0(x,t) = \psi_0(\omega_0,k)\, e^{ikx-i\omega_0 t}, \tag{S23}$$

where $\psi_0(\omega_0,k)$ is the eigenvector in Eq. (S19) for frequency ω_0. While the wavefield after the interface contains both forward- and backward-propagating components:

$$\Psi_1(x,t) = T\,\psi_1(\omega_1,k)\, e^{ikx-i\omega_1 t} + R\,\psi_1(\omega_1,k)\, e^{ikx+i\omega_1 t}. \tag{S24}$$

where $\psi_1(\omega_1,k)$ is the eigenvector in Eq. (S19) for frequency ω_1, T is the refracted coefficient, and R is the reflected coefficient. At the temporal interface $t=t_0$, the continuity conditions for displacement and velocity are:

$$\Psi_0 = \Psi_1, \quad \partial_t\Psi_0 = \partial_t\Psi_1. \tag{S25}$$

Substituting the waveforms into these conditions and evaluating at $t=t_0$, we obtain:

$$\begin{aligned}\psi_0(\omega_0,k) &= T\,\psi_1(\omega_1,k) + R\,\psi_1(\omega_1,k),\\ \omega_0\,\psi_0(\omega_0,k) &= \omega_1 T\,\psi_1(\omega_1,k) - \omega_1 R\,\psi_1(\omega_1,k).\end{aligned} \tag{S26}$$

We now take the inner product of both equations with the dual (left) eigenvector $\psi_1^\dagger(\omega_1,k)$. This yields:

$$\begin{aligned}\psi_1^\dagger(\omega_1,k)\psi_0(\omega_0,k) &= T+R,\\ \omega_0\psi_1^\dagger(\omega_1,k)\psi_0(\omega_0,k) &= \omega_1(T-R).\end{aligned} \tag{S27}$$

Solving this system of equations, we obtain the Fresnel-type refraction and reflection coefficients:

$$\begin{aligned}T &= \frac{\omega_1+\omega_0}{2\omega_0}\,\psi_1^\dagger(\omega_1,k)\psi_0(\omega_0,k),\\ R &= \frac{\omega_1-\omega_0}{2\omega_0}\,\psi_1^\dagger(\omega_1,k)\psi_0(\omega_0,k).\end{aligned} \tag{S28}$$

These expressions generalize the Fresnel equations to the Timoshenko beam case, where the reflection and refraction amplitudes are determined by both frequency shift and mode overlap across the temporal interface.

We now turn to the numerical evaluation of Snell's law and Fresnel coefficients using the Timoshenko beam model, and compare the results with those obtained from the Euler–Bernoulli beam model and COMSOL simulation. As shown in Fig. S13, the dispersion curves from all three models closely match, confirming the validity of the Euler–Bernoulli approximation. When the bending stiffness changes from $D/D_0 = 1$ to $D/D_0 = 0.85^2$, the normalized frequencies for the Timoshenko beam, Euler–Bernoulli beam, and COMSOL simulation change from $0.991, 0.989, 1$ to $0.844, 0.842, 0.85$, respectively. This yields corresponding ratios ω_1/ω_0 of 0.8517, 0.8514, and 0.850, respectively.

Moreover, the mode overlap term $\psi_1^\dagger(\omega,k)\psi_0(\omega,k)$, which appears in the Fresnel equations (Eq. S28), evaluates to 0.999999311089990, indicating that the mode shapes before and after switching remain nearly identical. Therefore, the reflection and refraction coefficients predicted by the Timoshenko and Euler–Bernoulli models are virtually indistinguishable. The differences are negligible in practical terms.

This agreement can be understood by introducing the characteristic shear length scale

$$\ell = \sqrt{\frac{EI}{\kappa GA}}, \tag{S29}$$

which leads to the dimensionless dynamic slenderness parameter $\Lambda = \ell k$. In our setup, $\Lambda \sim 10^{-4}$, indicating that shear effects are minimal and the Euler–Bernoulli model is valid.

To further validate this conclusion, we consider a more extreme case where the stiffness drops from $D/D_0 = 1$ to $D/D_0 = 0.25$ at the temporal interface. The normalized frequencies change from $0.991, 0.989, 1$ to $0.498, 0.497, 0.5$ for the Timoshenko beam, Euler–Bernoulli beam, and COMSOL, respectively, yielding $\omega_1/\omega_0 = 0.5025$, 0.5025, and 0.500. The mode overlap in this case is still very high: $\psi_1^\dagger(\omega,k)\psi_0(\omega,k) = 0.999998443354908$. Again, the Fresnel coefficients predicted by both beam models are nearly identical.

These results confirm that even under strong modulation, the Euler–Bernoulli beam theory provides sufficiently accurate predictions, and the effects of shear deformation and rotary inertia are negligible for our study. The small discrepancies observed are likely attributed to two factors: (i) incomplete termination of low-frequency incident signals before reaching the temporal boundary, and (ii) possible breakdown of the homogeneous beam assumption at high frequencies due to microstructural effects. These factors, rather than the beam model itself, account for the minor deviations between theory and experiment.

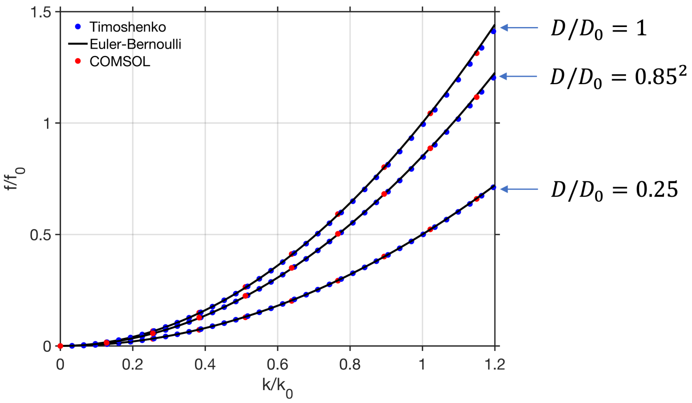

FIG. S13. Dispersion curves obtained from the Timoshenko beam model, the Euler–Bernoulli model, and COMSOL simulation. The top branch corresponds to the case with the circuit switch turned off. The bottom branches correspond to circuits with $R_1 = 5\,\mathrm{k}\Omega$ ($D/D_0 = 0.85^2$, used in the experiment) and $R_1 = 5.6\,\mathrm{k}\Omega$ ($D/D_0 = 0.25$, used in Fig. S7(a)).

We now turn to discuss the negligibility of other modes. In our study, the excitation frequency for temporal refraction and reflection is 6–10 kHz, which is significantly lower than the characteristic frequencies of shear modes ($\sim$776 kHz) and higher-order bending modes (above 80 kHz). The dispersion relations and mode shapes confirm that these modes lie far outside the excitation range. Moreover, the amplitude of the incident tone-burst signal drops below 0.01 at frequencies above 30 kHz, effectively suppressing the excitation of higher-order modes in practice. In experiments, a 1–40 kHz bandpass filter is also applied to further suppress potential high-frequency or non-flexural contributions.

Regarding longitudinal waves, they can in principle be excited by the piezoelectric actuator. However, the group velocity of the longitudinal mode is nearly eight times that of the fundamental flexural mode, meaning that any longitudinal wave passes through the time-varying segment well before the temporal modulation is active. Moreover, both the experimental and numerical signals focus on the transverse displacement $w(x,t)$; longitudinal displacement is neither measured in experiments nor extracted in simulations. Therefore, longitudinal waves do not affect the observed wavefields in either experiment or simulation. In experiments, a 1–40 kHz bandpass filter is also applied to further suppress potential high-frequency or non-flexural contributions.

Note on Damping Effects: In our system, damping arises from two sources: the elastic beam and the electrical circuits. For the beam, the intrinsic damping is characterized by a low material loss factor ($\eta \sim 10^{-4}$), resulting in negligible attenuation ($< 0.1\%$ amplitude loss over the beam length). For the circuit, effective damping depends on the resistor value R_0, which can introduce either loss or gain. By tuning $R_0 = 1$ MΩ, we balance these effects to minimize amplitude variation. As damping has minimal impact within the experimental timescales, it is omitted from the main theoretical model.

Note on Nonlinearity: In our study, both structural and circuit-level nonlinearities are negligible under the operating conditions. The maximum beam deflection is less than 0.001 mm—well below the geometric nonlinearity threshold ($w/h < 0.1$), and material strains remain within the elastic regime. Piezoelectric patches operate below $\pm$40 V, avoiding nonlinear dielectric behavior. On the circuit side, capacitors and resistors exhibit minimal voltage dependence, and operational amplifiers function well within their linear range. No harmonic distortion or waveform asymmetry is observed experimentally, and all FFT spectra agree with linear theory. Thus, the system behavior is accurately captured by a linear model, and the effect of nonlinearity can be safely ignored for the amplitude range used in this work.

12. NOETHER'S THEOREM AND CONSERVATION LAWS

A. Complex scalar field theory for the Euler-Bernoulli beam

As we know, when a flexural incident wave encounters an interface where the refractive index of the beam changes abruptly, it splits into a refracted wave and a reflected wave, as shown in Fig. S14a,b. Remarkably, the governing equation of the Euler-Bernoulli beam model inherently supports space-time duality, implying temporal analogs of reflection and refraction when a flexural wave encounters a time boundary, as illustrated in Fig. S14c,d. Wave scattering at both spatial and temporal boundaries adheres to Noether's theorem, which reveals the fundamental connection between the symmetries of a physical system and its conservation laws. In systems with abrupt spatial changes, the breakdown of space translation invariance leads to the non-conservation of momentum. However, the system remains invariant in the time direction, preserving time translation invariance and, consequently, the conservation of energy. In this section, energy and momentum are derived as Noether's charges in accordance with Noether's theorem.

In this paper, we examine wave propagation in the frequency domain, which involves complex analysis. There are two methods for deriving Noether's charge. The first method involves obtaining the expression for Noether's charge using real

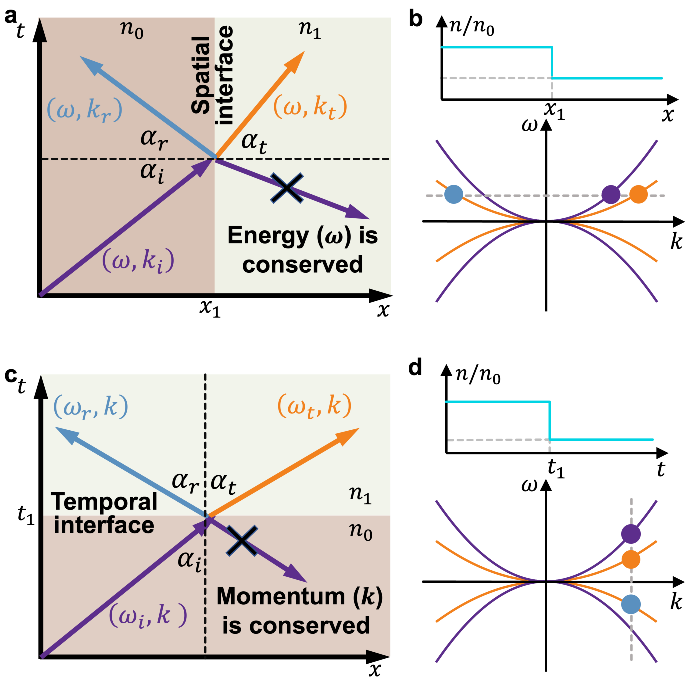

FIG. S14. **Refraction and reflection of flexural waves at a time interface.a** (**c**) Spacetime diagram of wave refraction and reflection at a spatial (temporal) interface. The subscripts i, r, and t correspond to the incident, reflected, and refracted waves, respectively. **b** (**d**) The top panel shows the index of refraction versus $x\,(t)$, with an abrupt change at $x_1\,(t_1)$. The bottom panel displays the dispersion curves: the medium before (after) $x_1\,(t_1)$ is represented by a purple (orange) solid line. The blue, red, and yellow points correspond to the incident, reflected, and refracted waves, respectively.

scalar field theory, followed by applying a periodic average to derive the corresponding charge in the complex domain [**?** **?**]. The second method derives the expression for Noether's charge directly from complex scalar field theory, avoiding time averaging. In this study, we employ the second method.

The complex scalar field is defined as $w(x,t) = [w_r(x,t) + iw_i(x,t)]/\sqrt{2}$, constructed from two identical real fields, $w_r(x,t)$ and $w_i(x,t)$ [**?** **?** **?**]. For convenience, we use $w(x,t)$ and $w^\dagger(x,t)$ as independent variables instead of $w_r(x,t)$ and $w_i(x,t)$. The Lagrangian for the Euler-Bernoulli beam can be extended from that of real scalar field theory [**?**] as follows:

$$\mathcal{L} = \frac{1}{2}\rho A \partial_t w^\dagger(x,t)\partial_t w(x,t) - \frac{1}{2}EI\partial_{xx}w^\dagger(x,t)\partial_{xx}w(x,t) \tag{S30}$$

And the action S, the time integral of the Lagrangian $\mathcal{L}$, can be written as

$$S = \int \mathcal{L}dt = \int \mathcal{L}\left(w_t, w_t^\dagger, w_{xx}, w_{xx}^\dagger\right) dxdt \tag{S31}$$

The principle of least action states [**?**]

$$\begin{aligned}
0 &= \delta S \\
&= \int dxdt \Bigg\{ \frac{\partial \mathcal{L}}{\partial(\partial_t w)}\delta(\partial_t w) + \frac{\partial \mathcal{L}}{\partial(\partial_{xx} w)}\delta(\partial_{xx} w) + \frac{\partial \mathcal{L}}{\partial(\partial_t w^\dagger)}\delta(\partial_t w^\dagger) + \frac{\partial \mathcal{L}}{\partial(\partial_{xx} w^\dagger)}\delta(\partial_{xx} w^\dagger) \Bigg\} \\
&= \int dxdt \Bigg\{ \partial_t\left(\frac{\partial \mathcal{L}}{\partial(\partial_t w)}\delta w\right) - \partial_t\left(\frac{\partial \mathcal{L}}{\partial(\partial_t w)}\right)\delta w + \partial_x\left(\frac{\partial \mathcal{L}}{\partial(\partial_{xx} w)}\partial_x \delta w\right) - \partial_x\left(\partial_x\frac{\partial \mathcal{L}}{\partial(\partial_{xx} w)}\delta w\right) + \partial_{xx}\left(\frac{\partial \mathcal{L}}{\partial(\partial_{xx} w)}\right)\delta w \\
&\quad + \partial_t\left(\frac{\partial \mathcal{L}}{\partial(\partial_t w^\dagger)}\delta w^\dagger\right) - \partial_t\left(\frac{\partial \mathcal{L}}{\partial(\partial_t w^\dagger)}\right)\delta w^\dagger + \partial_x\left(\frac{\partial \mathcal{L}}{\partial(\partial_{xx} w^\dagger)}\partial_x \delta w^\dagger\right) - \partial_x\left(\partial_x\frac{\partial \mathcal{L}}{\partial(\partial_{xx} w^\dagger)}\delta w^\dagger\right) + \partial_{xx}\left(\frac{\partial \mathcal{L}}{\partial(\partial_{xx} w^\dagger)}\right)\delta w^\dagger \Bigg\} \\
&= \int dxdt \Bigg\{ -\partial_t\left(\frac{\partial \mathcal{L}}{\partial(\partial_t w)}\right)\delta w + \partial_{xx}\left(\frac{\partial \mathcal{L}}{\partial(\partial_{xx} w)}\right)\delta w - \partial_t\left(\frac{\partial \mathcal{L}}{\partial(\partial_t w^\dagger)}\right)\delta w^\dagger + \partial_{xx}\left(\frac{\partial \mathcal{L}}{\partial(\partial_{xx} w^\dagger)}\right)\delta w^\dagger \Bigg\}
\end{aligned} \tag{S32}$$

where the rules of variational operations can be found in [**?**]. By factoring out δw ($\delta w^\dagger$) from the first two terms (last two terms), we note that, since the integral must vanish for arbitrary δw ($\delta w^\dagger$), the quantity multiplying δw ($\delta w^\dagger$) must

also vanish at all points. This leads us to the Euler-Lagrange equation of motion for the complex field:

$$\partial_t\left(\frac{\partial\mathcal{L}}{\partial\left(\partial_t w\right)}\right)-\partial_{xx}\left(\frac{\partial\mathcal{L}}{\partial\left(\partial_{xx} w\right)}\right)=0 \tag{S33}$$

$$\partial_t\left(\frac{\partial\mathcal{L}}{\partial\left(\partial_t w^\dagger\right)}\right)-\partial_{xx}\left(\frac{\partial\mathcal{L}}{\partial\left(\partial_{xx} w^\dagger\right)}\right)=0. \tag{S34}$$

B. Time translation symmetry and energy conservation

We can describe the infinitesimal translation of time as

$$t\rightarrow t+\delta t \tag{S35}$$

alternatively as a transformation of the field configuration

$$\begin{aligned} w(x,t)&\rightarrow w(x,t)=w(x,t)+\delta t\partial_t w(x,t),\\ w^\dagger(x,t)&\rightarrow w^\dagger(x,t)=w^\dagger(x,t)+\delta t\partial_t w^\dagger(x,t). \end{aligned} \tag{S36}$$

More generally, we can allow the action to change by a surface term, since the presence of such a term would not affect our derivation of the Euler-Lagrange equations of motion Eq. (S33,S34). The Lagrangian, therefore, must be invariant under Eq. (S35) up to a divergence:

$$\mathcal{L}\rightarrow\mathcal{L}+\delta t\partial_t\mathcal{L}. \tag{S37}$$

On the other hand, the change in the Lagrangian density $\mathcal{L}$ is then given by

$$\begin{aligned} \delta\mathcal{L}&=\frac{\partial\mathcal{L}}{\partial\left(\partial_t w\right)}\delta\left(\partial_t w\right)+\frac{\partial\mathcal{L}}{\partial\left(\partial_t w^\dagger\right)}\delta\left(\partial_t w^\dagger\right)+\frac{\partial\mathcal{L}}{\partial\left(\partial_{xx} w\right)}\delta\left(\partial_{xx} w\right)+\frac{\partial\mathcal{L}}{\partial\left(\partial_{xx} w^\dagger\right)}\delta\left(\partial_{xx} w^\dagger\right)\\ &=\frac{\partial\mathcal{L}}{\partial\left(\partial_t w\right)}\partial_t\left(\delta w\right)+\frac{\partial\mathcal{L}}{\partial\left(\partial_t w^\dagger\right)}\partial_t\left(\delta w^\dagger\right)+\frac{\partial\mathcal{L}}{\partial\left(\partial_{xx} w\right)}\partial_{xx}\left(\delta w\right)+\frac{\partial\mathcal{L}}{\partial\left(\partial_{xx} w^\dagger\right)}\partial_{xx}\left(\delta w^\dagger\right)\\ &=\delta t\left[\frac{\partial\mathcal{L}}{\partial\left(\partial_t w\right)}\partial_t\left(w_t\right)+\frac{\partial\mathcal{L}}{\partial\left(\partial_t w^\dagger\right)}\partial_t\left(w_t^\dagger\right)+\frac{\partial\mathcal{L}}{\partial\left(\partial_{xx} w\right)}\partial_{xx}\left(w_t\right)+\frac{\partial\mathcal{L}}{\partial\left(\partial_{xx} w^\dagger\right)}\partial_{xx}\left(w_t^\dagger\right)\right] \end{aligned} \tag{S38}$$

Multiplying Eq. (S33) with $\delta t w_t$ and Eq. (S34) with $\delta t w_t^\dagger$, and adding them into Eq. (S38) gives

$$\begin{aligned} \delta\mathcal{L}=&\,\delta t\partial_t\left(\frac{\partial\mathcal{L}}{\partial\left(\partial_t w\right)}w_t+\frac{\partial\mathcal{L}}{\partial\left(\partial_t w^\dagger\right)}w_t^\dagger\right)\\ &+\delta t\partial_x\left[\partial_x\left(\frac{\partial\mathcal{L}}{\partial\left(\partial_{xx} w\right)}\right)w_t+\partial_x\left(\frac{\partial\mathcal{L}}{\partial\left(\partial_{xx} w^\dagger\right)}\right)w_t^\dagger+\frac{\partial\mathcal{L}}{\partial\left(\partial_{xx} w\right)}w_{xt}+\frac{\partial\mathcal{L}}{\partial\left(\partial_{xx} w^\dagger\right)}w_{xt}^\dagger\right] \end{aligned} \tag{S39}$$

The Lagrangian density $\mathcal{L}$ could very well change by a divergence $\delta\mathcal{L}=\delta t\partial_t\mathcal{L}$. Therefore, the combination of Eq. (S39) and Eq. (S37) upon arbitrary infinitesimal time translation δt leads to

$$\partial_t j^{tt}+\partial_x j^{tx}=0, \tag{S40}$$

where

$$\begin{aligned} j^{tt}&=\frac{\partial\mathcal{L}}{\partial\left(\partial_t w\right)}w_t+\frac{\partial\mathcal{L}}{\partial\left(\partial_t w^\dagger\right)}w_t^\dagger-\mathcal{L}\\ j^{tx}&=\partial_x\left(\frac{\partial\mathcal{L}}{\partial\left(\partial_{xx} w\right)}\right)w_t+\partial_x\left(\frac{\partial\mathcal{L}}{\partial\left(\partial_{xx} w^\dagger\right)}\right)w_t^\dagger+\frac{\partial\mathcal{L}}{\partial\left(\partial_{xx} w\right)}w_{xt}+\frac{\partial\mathcal{L}}{\partial\left(\partial_{xx} w^\dagger\right)}w_{xt}^\dagger \end{aligned} \tag{S41}$$

Then, performing the integration over x at constant time gives

$$\partial_t\int j^{tt}dx=0, \tag{S42}$$

where $\int\partial_x j^{tx}dx=0$ is used because this will vanish if the line is long enough [?]. Now we define $\mathcal{H}$ as the energy density:

$$\mathcal{H}=\frac{\partial\mathcal{L}}{\partial\left(\partial_t w\right)}\partial_t w+\frac{\partial\mathcal{L}}{\partial\left(\partial_t w^\dagger\right)}\partial_t w^\dagger-\mathcal{L} \tag{S43}$$

And the Hamiltonian (energy)

$$H = \int \mathcal{H} dx = \int dx \left(\frac{1}{2}\rho A \partial_t w^\dagger(x,t)\partial_t w(x,t) + \frac{1}{2} EI \partial_{xx} w^\dagger(x,t)\partial_{xx} w(x,t) \right). \tag{S44}$$

is conserved for system with time translation symmetry according to Eq. (S42). The energy before the switching event is

$$H_0 = \left(\frac{1}{2}\rho A \omega_0^2 + \frac{1}{2} E_0 I_0 k_0^4 \right) A_i^2 = \frac{1}{Z_0^2}\rho A A_i^2, \tag{S45}$$

whereas the energy density after the switching event is

$$H_1 = \left(\frac{1}{2}\rho A \omega_1^2 + \frac{1}{2} E_1 I_1 k_1^4 \right) \left(T^2 + R^2\right) A_i^2 = \frac{Z_0^2 + Z_1^2}{2Z_1^4}\rho A A_i^2 = \frac{Z_0^4 + Z_0^2 Z_1^2}{2Z_1^4} H_0, \tag{S46}$$

where Eq. (9) in the main text is used. Now we can be easily verified that the energy in Eq. (S46) is not conserved:

$$H_1 \neq H_0 \tag{S47}$$

for temporal media with $Z_0 \neq Z_1$. We conclude that the breaking of time translation symmetry leads to the breakdown of the conservation of energy.

C. Space translation symmetry and momentum conservation

We can describe the infinitesimal translation of time as

$$x \to x + \delta x \tag{S48}$$

alternatively as a transformation of the field configuration

$$\begin{aligned} w(x,t) &\to w(x,t) = w(x,t) + \delta x \partial_x w(x,t), \\ w^\dagger(x,t) &\to w^\dagger(x,t) = w^\dagger(x,t) + \delta x \partial_x w^\dagger(x,t). \end{aligned} \tag{S49}$$

More generally, we can allow the action to change by a surface term, since the presence of such a term would not affect our derivation of the Euler-Lagrange equations of motion Eq. (S33,S34). The Lagrangian, therefore, must be invariant under Eq. (S48) up to a divergence:

$$\mathcal{L} \to \mathcal{L} + \delta x \partial_x \mathcal{L}. \tag{S50}$$

On the other hand, the change in the Lagrangian density $\mathcal{L}$ is then given by

$$\begin{aligned} \delta\mathcal{L} &= \frac{\partial \mathcal{L}}{\partial(\partial_t w)}\delta(\partial_t w) + \frac{\partial \mathcal{L}}{\partial(\partial_t w^\dagger)}\delta\left(\partial_t w^\dagger\right) + \frac{\partial \mathcal{L}}{\partial(\partial_{xx} w)}\delta(\partial_{xx} w) + \frac{\partial \mathcal{L}}{\partial(\partial_{xx} w^\dagger)}\delta\left(\partial_{xx} w^\dagger\right) \\ &= \delta x \left[\frac{\partial \mathcal{L}}{\partial(\partial_t w)}\partial_t(w_x) + \frac{\partial \mathcal{L}}{\partial(\partial_t w^\dagger)}\partial_t\left(w_x^\dagger\right) + \frac{\partial \mathcal{L}}{\partial(\partial_{xx} w)}\partial_{xx}(w_x) + \frac{\partial \mathcal{L}}{\partial(\partial_{xx} w^\dagger)}\partial_{xx}\left(w_x^\dagger\right) \right] \end{aligned} \tag{S51}$$

Multiplying Eq. (S33) with $\delta x w_x$ and Eq. (S34) with $\delta x w_x^\dagger$, and adding them into Eq. (S51) gives

$$\begin{aligned} \delta\mathcal{L} &= \delta x \partial_t \left(\frac{\partial \mathcal{L}}{\partial(\partial_t w)} w_x + \frac{\partial \mathcal{L}}{\partial(\partial_t w^\dagger)} w_x^\dagger \right) \\ &+ \delta x \partial_x \left[\partial_x \left(\frac{\partial \mathcal{L}}{\partial(\partial_{xx} w)} \right) w_x + \partial_x \left(\frac{\partial \mathcal{L}}{\partial(\partial_{xx} w^\dagger)} \right) w_x^\dagger + \frac{\partial \mathcal{L}}{\partial(\partial_{xx} w)} w_{xx} + \frac{\partial \mathcal{L}}{\partial(\partial_{xx} w^\dagger)} w_{xx}^\dagger \right] \end{aligned} \tag{S52}$$

The Lagrangian density $\mathcal{L}$ may change by a divergence, $\delta\mathcal{L} = \delta x \partial_x \mathcal{L}$. Therefore, combining Eq. (S51) and Eq. (S50) under an arbitrary infinitesimal time translation δx results in the following expression:

$$\partial_t j^{tx} + \partial_x j^{xx} = 0, \tag{S53}$$

where

$$\begin{aligned} j^{tx} &= \frac{\partial \mathcal{L}}{\partial(\partial_t w)} w_x + \frac{\partial \mathcal{L}}{\partial(\partial_t w^\dagger)} w_x^\dagger \\ j^{xx} &= \partial_x \left(\frac{\partial \mathcal{L}}{\partial(\partial_{xx} w)} \right) w_x + \partial_x \left(\frac{\partial \mathcal{L}}{\partial(\partial_{xx} w^\dagger)} \right) w_x^\dagger + \frac{\partial \mathcal{L}}{\partial(\partial_{xx} w)} w_{xx} + \frac{\partial \mathcal{L}}{\partial(\partial_{xx} w^\dagger)} w_{xx}^\dagger \end{aligned} \tag{S54}$$

Then, performing the integration over x at constant time gives

$$\partial_t \int j^{tx} dx = 0, \tag{S55}$$

where $\int \partial_x j^{xx} dx = 0$ is used because this will vanish if the line is long enough [?]. Now we define p as the momentum density:

$$p = j^{tx} = \frac{\partial \mathcal{L}}{\partial\left(\partial_t w\right)} \partial_x w + \frac{\partial \mathcal{L}}{\partial\left(\partial_t w^{\dagger}\right)} \partial_x w^{\dagger} \tag{S56}$$

The conserved charges (momentum) associated with spatial translations are

$$P = \int p dx = \int dx \rho A[\left(\partial_t w^{\dagger}\right) w_x + \left(\partial_t w\right) w_x^{\dagger}]. \tag{S57}$$

The momentum of the wave before the switching event is

$$P_0 = 2\rho A \omega_0 k_0 A_i^2, \tag{S58}$$

whereas the momentum of waves after the switching event is

$$P_1 = 2\rho A \omega_1 k_1 (T^2 - R^2) A_i^2. \tag{S59}$$

With the aid of Eq. (9) in the main text, the momentum conservation can be easily verified that

$$P_0 = P_1 = 2 Z_0 k_0^3 A_i^2. \tag{S60}$$

13. OPTIMIZATION METHOD FOR BROADBAND ANTI-REFLECTION AND WAVE AMPLIFICATION AT MULTIPLE TIME INTERFACES

In this section, we provide a detailed description of the optimization problem for designing temporal multilayer media aimed at eliminating broadband reflected waves and enhancing wave amplification.

For single-frequency reflected wave elimination, the optimization problem can be proposed as

$$\begin{aligned} \underset{n_1, t_2}{\text{minimize}} \quad & |R(n_1, t_2, f_0 = 6 \text{ kHz})|^2 \\ \text{subject to} \quad & n_0 \leq n_1 \leq 2n_0, \\ & \Delta t_1 > 0 \end{aligned} \tag{S61}$$

For broadband reflected wave elimination, the optimization problem can be proposed as

$$\begin{aligned} \underset{\boldsymbol{n}, \boldsymbol{t}}{\text{minimize}} \quad & \textstyle\int_{f_a}^{f_b} |R(\boldsymbol{n}, \boldsymbol{t}), f_0|^2 df_0 \\ \text{subject to} \quad & \min(n_0, n_{M+1}) \leq n_i \leq \max(n_0, n_{M+1}), \quad i = 1, \ldots, M \\ & \Delta t_j > 0, \quad j = 1, \ldots, M \end{aligned} \tag{S62}$$

where $f_a = 0.5 f_0$, $f_b = 1.5 f_0$, $M = 4$, $n_{M+1} = 2n_0$.

For wave amplification, the optimization problem can be proposed as

$$\begin{aligned} \underset{\boldsymbol{n}, \boldsymbol{t}}{\text{minimize}} \quad & -|T(\boldsymbol{n}, \boldsymbol{t}, f_0 = 6 \text{ kHz})|^2 \\ \text{subject to} \quad & 0 \leq n_i \leq 3n_0, \quad i = 1, \ldots, M \\ & \Delta t_j > 0, \quad j = 1, \ldots, M \end{aligned} \tag{S63}$$

where $T(\boldsymbol{n}, \boldsymbol{t})$ is the refraction coefficient, $M = 3$, $n_{M+1} = n_0$.

The constrained nonlinear optimization problem is solved numerically by calling the MATLAB function "`fmincon`" with given random initial conditions satisfying the constraints, where the sequential quadratic programming (SQP) algorithm is implemented.

14. RESISTOR FUNCTIONS IN SMART WAVEFORM MORPHING AND INFORMATION

In the main text, the transfer functions can be modulated into various forms, such as sinusoidal functions and smooth step functions for smart waveform morphing and information coding. The sinusoidal function of the resistor in Fig. 6b of the main text is

$$R_1(t) = 5.3 + 0.1 \cos \frac{2\pi t}{T} \tag{S64}$$

where $T = 0.2$ s. The smooth step function of the resistor in Fig. 6e of the main text is

$$R_1(t) = \begin{cases} 5.2 & 0 \le t \le \frac{T-\Delta T}{2} \\ 0.2 \times \frac{t-mT/2+\Delta T/2}{\Delta T} + 5.2 & \frac{mT-\Delta T}{2} \le t \le \frac{mT+\Delta T}{2} \\ 5.4 & \frac{mT+\Delta T}{2} \le t \le mT - \frac{\Delta T}{2} \\ -0.2 \times \frac{t-mT+\Delta T/2}{\Delta T} + 5.4 & mT - \frac{\Delta T}{2} \le t \le mT + \frac{\Delta T}{2} \\ 5.2 & mT + \frac{\Delta T}{2} \le t \le \frac{3mT}{2} - \frac{\Delta T}{2} \\ 0.2 \times \frac{t-3mT/2+\Delta T/2}{\Delta T} + 5.2 & \frac{3mT-\Delta T}{2} \le t \le \frac{3mT+\Delta T}{2} \end{cases}, \quad m = 1, 2, 3..., \tag{S65}$$

where $T = 0.2$ s and $\Delta T = 0.02$ s. The smooth step function of the resistor in Fig. 7b of the main text is

$$R_1(t) = \begin{cases} 5.2 & 0 \le t \le \frac{2T-\Delta T}{2} \\ 0.2 \times \frac{t-T+\Delta T/2}{\Delta T} + 5.2 & \frac{T-\Delta T}{2} \le t \le \frac{2T+\Delta T}{2} \\ 5.4 & \frac{2T+\Delta T}{2} \le t \le \frac{3T-\Delta T}{2} \\ -0.08 \times \frac{t-3T+\Delta T/2}{\Delta T} + 5.4 & \frac{3T-\Delta T}{2} \le t \le 2T + \frac{\Delta T}{2} \\ 5.32 & 2T + \frac{\Delta T}{2} \le t \le \frac{5T}{2} - \frac{\Delta T}{2} \\ -0.12 \times \frac{t-5T/2+\Delta T/2}{\Delta T} + 5.32 & \frac{5T-\Delta T}{2} \le t \le \frac{5T+\Delta T}{2} \\ 5.2 & \frac{5T-\Delta T}{2} \le t \le 3T \end{cases}, \tag{S66}$$

where $T = 0.2$ s and $\Delta T = 0.02$ s. The values 5.2 kΩ, 5.3 kΩ, and 5.4 kΩ were chosen based on a balance between functional performance and system stability:

- **5.2 kΩ** marks the point at which the effective bending stiffness begins to become negative, which leads to significant amplitude gain and thus is useful for distinguishing the "dash" signal.
- **5.4 kΩ** is the highest resistance we can apply before the system becomes unstable due to excessive feedback-induced amplification in the circuit.
- **5.3 kΩ** serves as a middle setting that produces a clearly distinguishable amplitude between the 5.2 and 5.4 kΩ cases, and is assigned to the "dot" signal in Morse coding.
